# Myeloid Knockout of HIF-1****α**** Does Not Markedly Affect Hemorrhage/Resuscitation-Induced Inflammation and Hepatic Injury

**DOI:** 10.1155/2014/930419

**Published:** 2014-06-01

**Authors:** G. Wetzel, B. Relja, A. Klarner, D. Henrich, N. Dehne, B. Brühne, M. Lehnert, I. Marzi

**Affiliations:** ^1^Department of Trauma, Hand and Reconstructive Surgery, University Hospital Frankfurt, Goethe University, 60590 Frankfurt, Germany; ^2^Institute of Biochemistry I/ZAFES, Goethe University, 60590 Frankfurt, Germany

## Abstract

*Background*. Hypoxia-inducible factor-1**α** (HIF-1**α**) and NF-**κ**B play important roles in the inflammatory response after hemorrhagic shock and resuscitation (H/R). Here, the role of myeloid HIF-1**α** in liver hypoxia, injury, and inflammation after H/R with special regard to NF-**κ**B activation was studied. *Methods*. Mice with a conditional HIF-1**α** knockout (KO) in myeloid cell-line and wild-type (WT) controls were hemorrhaged for 90 min (30 ± 2 mm Hg) and resuscitated. Controls underwent only surgical procedures. *Results*. After six hours, H/R enhanced the expression of HIF-1**α**-induced genes vascular endothelial growth factor (VEGF) and adrenomedullin (ADM). In KO mice, this was not observed. H/R-induced liver injury in HIF-1**α** KO was comparable to WT. Elevated plasma interleukin-6 (IL-6) levels after H/R were not reduced by HIF-1**α** KO. Local hepatic hypoxia was not significantly reduced in HIF-1**α** KO compared to controls after H/R. H/R-induced NF-**κ**B phosphorylation in liver did not significantly differ between WT and KO. *Conclusions*. Here, deleting HIF-1**α** in myeloid cells and thereby in Kupffer cells was not protective after H/R. This data indicates that other factors, such as NF-**κ**B, due to its upregulated phosphorylation in WT and KO mice, contrary to HIF-1**α**, are rather key modulators of inflammation after H/R in our model.

## 1. Introduction

Trauma accounts for 10% of deaths worldwide, with blood loss as the major contributor to mortality after trauma [1–4]. In patients who survive the hemorrhagic shock and become resuscitated (H/R) a systemic inflammatory response syndrome (SIRS) may develop in the early posttraumatic course. SIRS can cause a multiple organ dysfunction syndrome (MODS) resulting in increase of posttraumatic mortality rates up to 60% [2–5]. H/R compromises the microcirculatory integrity with subsequent activation of immune cells (monocytes and leukocytes) inducing their hyperresponsiveness to pathogens and/or inflammatory stimuli [6, 7]. Here, activated immune cells release proinflammatory mediators as well as reactive oxygen species (ROS) and thereby promote cellular membrane, DNA as well as protein damage, and finally tissue injury [8, 9]. Increased proinflammatory cytokine levels are closely associated with increased liver damage and mortality rates in H/R models [10–13].

Adaptation to hypoxic conditions in cells is mainly mediated by hypoxia-inducible factor (HIF). Under well-oxygenated conditions HIF-1*α* becomes hydroxylated, thereby generating a binding site for the von Hippel-Lindau tumor suppressor protein, a component of a ubiquitin ligase complex [14, 15]. The polyubiquitinated HIF-1*α* is then degraded and does not interact with the DNA [14, 16]. Exposing cells to hypoxia increases the expression of HIF-1*α* which can translocate to the nucleus, dimerize with HIF-1*β*, and induce transcription of numerous hypoxia-related genes, thereby enabling cells to adapt to hypoxic conditions [17, 18]. It is well established that HIF-1 is important for activated immune cells by maintaining ATP levels and allowing induction of inflammation relevant genes like iNOS or CRAMP [19, 20].

Next to HIF as a key player involved in the regulation of hypoxic inflammation, another transcription factor the nuclear factor-kappaB (NF-*κ*B) demonstrated its central role in this setting. Both transcription factors display different degrees activation during hypoxia. Moreover, their interdependence has been postulated. NF-*κ*B is involved in the regulation of inflammatory and immune responses and is activated in the liver after H/R [21]. Hypoxia, ROS, and various cytokines induce NF-*κ*B activation [22, 23]. Upregulated gene expression of proinflammatory mediators such as IL-6 is closely associated with NF-*κ*B signaling and hepatic injury after H/R [24–26].

Nevertheless, the intracellular pathways involved in H/R-induced inflammatory changes and hepatic injury are not well characterized yet. A decisive implication of both pathways and transcription factors HIF-1*α* and NF-*κ*B in H/R conditions has been described. Moreover, both factors may interfere [27]. A crosstalk between NF-*κ*B and HIF has recently been described as responsible element for basal HIF-1*α* gene expression [28]. On the other hand, HIF overexpression interferes with NF-*κ*B signaling by increasing NF-*κ*B activity and gene expression [29].

Accordingly, we studied the influence of HIF-1*α* and the role of NF-*κ*B in the pathogenesis of hepatic injury and inflammation induced by H/R.

## 2. Material and Methods

### 2.1. Animals and Experimental Model

Mice with a conditional HIF-1*α* knockout of the myeloid cell-line were kindly provided by Professor Dr. Brühne (Institute of Biochemistry, Goethe University, Frankfurt, Germany) and bred in our in-house facility. Mice were created by targeted deletions of Exon 2 of the HIF-1*α* gene by crossing double-floxed mice into a background of Cre expression driven by the lysozyme M promoter (lysMcre), which allows specific deletion of HIF-1*α* in the myeloid lineage as described [20]. Wild-type littermate without HIF-1*α* deletion was taken as control.

Female mice (8–12 weeks, 20–25 g) were randomly divided into shock (H/R) or sham group. Both sham groups implied six animals, WT-H/R group eight animals, and HIF-KO-H/R group 12 animals. Anaesthesia was performed with isoflurane (2%), and both femoral arteries were cannulated with polyethylene tubing. Shock was induced over 5 min by withdrawing blood into a heparinized syringe (10 U) to a mean arterial blood pressure of 30 ± 2 mm Hg. Arterial pressure was monitored and recorded using a blood pressure analyzer (BPA 400, Digi-Med, Louisville, KY). After 90 min mice were resuscitated by transfusion of 60% of the shed blood plus a volume of Ringer's solution corresponding to 50% of the shed blood volume over 30 min. After resuscitation, catheters were removed, the vessels were occluded, the incisions were flushed with lidocaine, and the wounds were closed. Sham-operated animals underwent the same surgical procedures including catheterization of both femoral arteries but hemorrhage was not carried out. Six hours after the end of resuscitation, the animals were reanesthetized for sacrifice. The* vena cava *was punctured, blood was collected, and liver tissue was harvested. For each mouse, the two right dorsal liver lobes were snap-frozen in liquid nitrogen. The remaining liver was flushed with normal saline, then fixed with 10% buffered formalin through the portal vein, embedded in paraffin, and subsequently sectioned and stained with haematoxylin-eosin. Body temperature was measured with a rectal temperature sensor and maintained between 35 and 37°C throughout the experiment with a heating pad. Animal protocols were approved by the Veterinary Department of the Regional Council in Darmstadt, Germany.

### 2.2. Tissue Hypoxia Evaluation

Intrahepatic hypoxia was marked* in vivo*, using pimonidazole hydrochloride Hypoxyprobe-1 (Omni-Kit, Burlington, USA), a 2-nitroimidazole immunochemical hypoxia marker, according to the manufacturer's instructions. With decreasing pO_2_ below 10 mm Hg, the 2-nitroimidazole irreversibly binds to the thiol groups in proteins to form intracellular adducts that can be detected by antibodies. In brief, equal doses of 60 mg/kg body weight of Hypoxyprobe-1 in a 0.5 mL (0.7%) NaCl bolus were injected intraperitoneally into three animals from each experimental group 1 h prior to sacrifice. After the sacrifice livers were immediately removed and placed in 4% formalin, to be used for immunohistochemical staining as described later.

### 2.3. Examination of Tissue Injury

Plasma was stored at −80°C for later analysis of alanine aminotransferase (ALT) and lactate dehydrogenase (LDH) using the Spotchem EZ SP-4430 device (Arkray, Japan). Determination of histological damage was performed by an independent examiner who allocated the hematoxylin-eosin stained liver sections to the various experimental groups in a blinded manner as published previously [21].

### 2.4. Detection of Hepatic Tissue Hypoxia

Livers removed from mice injected with Hypoxyprobe-1 were embedded in paraffin and sectioned. After deparaffinization subsequent gradual hydration in serial ethanol dilutions and quenching of tissue peroxidase with 3% hydrogen peroxide followed. The antigen retrieval was performed in a 10 mM phosphate buffer in a steamer device. The slides were incubated with the primary antibody (PAb2627AP purified rabbit antibody, diluted 1 : 50) for 30 min, repeatedly rinsed, and again incubated for 30 min with the secondary donkey anti-rabbit IgG-Cy3 antibody (1 : 50). Sections were captured at 400x and analyzed in a blinded manner. The extent of labeling in the liver tissue was defined as the percentage of the field area within a preset color range determined by the software (Adobe Photoshop 7.0). Data from each tissue section (10 fields per section) were pooled to determine mean values.

### 2.5. Quantification of Cytokine Levels

Concentrations of plasma and hepatic IL-6 were determined using a Quantikine Mouse IL-6 ELISA kit of R&D Systems according to the manufacturer's instructions (Wiesbaden-Nordenstadt, Germany). ELISA was performed using Infinite M200 microplate reader (Tecan, Männedorf, Switzerland).

### 2.6. Ribonucleic Acid (RNA) Isolation and Quantitative Reverse-Transcription-Polymerase Chain Reaction (RT-PCR)

Total RNA of snap-frozen liver lobes was isolated using the RNeasy system (Qiagen, Hilden, Germany) according to the manufacturer's instructions. The residual amounts of DNA remaining were removed using the RNase-Free DNase Set according to the manufacturer's instructions (Qiagen, Hilden, Germany). The RNA was stored immediately at −80°C. Quality and amount of the RNA were determined photometrically using the NanoDrop ND-1000 device (NanoDrop Technologies, Wilmington, DE, USA). RNA was subsequently reversely transcribed using the AffinityScript QPCR-cDNA synthesis kit (Stratagene, La Jolla, CA, USA) following the manufacturer's instructions and used for qPCR. To determine the mRNA expression of Vegf and Adm, qPCR was carried out on a Stratagene MX3005p QPCR system (Stratagene) using gene-specific primers for mouse Vegf (NM_001025250, UniGene#: Mm.282184, and Cat#: PPM03041E) and mouse Adm (NM_009627, UniGene#: Rn.256342, and Cat#: PPM05207A) purchased from Qiagen (Qiagen, Hilden, Germany). As reference gene, the expression of 18srRNA with mouse 18srRNA (Cat#: PPM57735E, Qiagen, Hilden, Germany) was measured. Sequences of these primers are not available. PCR reaction was set up with 1x RT2 SYBR Green/Rox qPCR Master mix (SABiosciences) in a 25 *μ*L volume according to the manufacturer's instructions. A two-step amplification protocol consisting of initial denaturation at 95°C for 10 min followed by 40 cycles with 15 s denaturation at 95°C and 60 s annealing/extension at 60°C was chosen. A melting-curve analysis was applied to control the specificity of amplification products. Relative expression of each target gene mRNA level was then calculated using the comparative threshold-cycle (CT) method (2^−ΔΔCT^ method). In brief, the amount of target mRNA in each sample was normalized to the amount of 18srRNA mRNA to give ΔCT. The relative mRNA expression of target genes is presented as fold increase calculated after normalization to 18srRNA. RT-PCR was performed as described before [30].

### 2.7. Western Blotting for Intracellular Signalling

Liver tissue was homogenized in lysis buffer at 4°C, followed by centrifugation for 30 min at 4°C at 20.000 g. Supernatants were stored at −80°C for later analysis. Lysates (50 *μ*g protein) were separated by electrophoresis on 12% polyacrylamide SDS gels and transferred to nitrocellulose membranes (Amersham-Buchler, Braunschweig, Germany). NF-*κ*B (phospho) was detected using rabbit monoclonal Phospho-NF-*κ*B p65 (Ser536) antibody and NF-*κ*B using rabbit monoclonal NF-*κ*B p65 antibody, respectively (Cell Signaling). Determination of *β*-actin with anti-*β*-actin antibody (Sigma, Taufkirchen, Germany) served as a loading control. Blots were blocked (10% nonfat dry milk in 1 mM Tris, 150 mM NaCl, pH 7.4) for 1 h, incubated for 1 h at RT with primary antibody (diluted according to the manufacturer's instructions in blocking buffer with 0.5% tween 20 and 0.5% BSA), and then incubated for 1 h with horseradish peroxidase-conjugated secondary antibody (Santa Cruz Biotechnology, Santa Cruz, CA, USA) diluted 1 : 1000 in blocking buffer with 0.5% tween 20 and 0.5% bovine serum albumin at RT. Proteins were detected with ECL western blot detection reagents (GE Healthcare, Munich, Germany). Western blot was performed as described previously [31]. Individual bands were semiquantified by densitometric measurements and the activation state of NF-*κ*B p65 was calculated as the ratio of phosphorylated and total protein values of densitometry data in per cent as described previously [30].

### 2.8. Statistical Analysis

Differences between groups were determined by one-way analysis of variance (ANOVA) using a multiple comparison procedure (Student-Newman-Keuls* post hoc*). Changes in target gene expression were analyzed by Wilcoxon matched-pair analysis followed by Bonferroni correction. A *P* value of less than 0.05 was considered significant. Data are given as mean ± standard error of the mean. All statistical analyses were performed employing GraphPad Prism 5 (GraphPad Software, Inc., San Diego, CA).

## 3. Results

### 3.1. Hemodynamic Characteristics of Hemorrhage and Resuscitation

**Figure 1:**
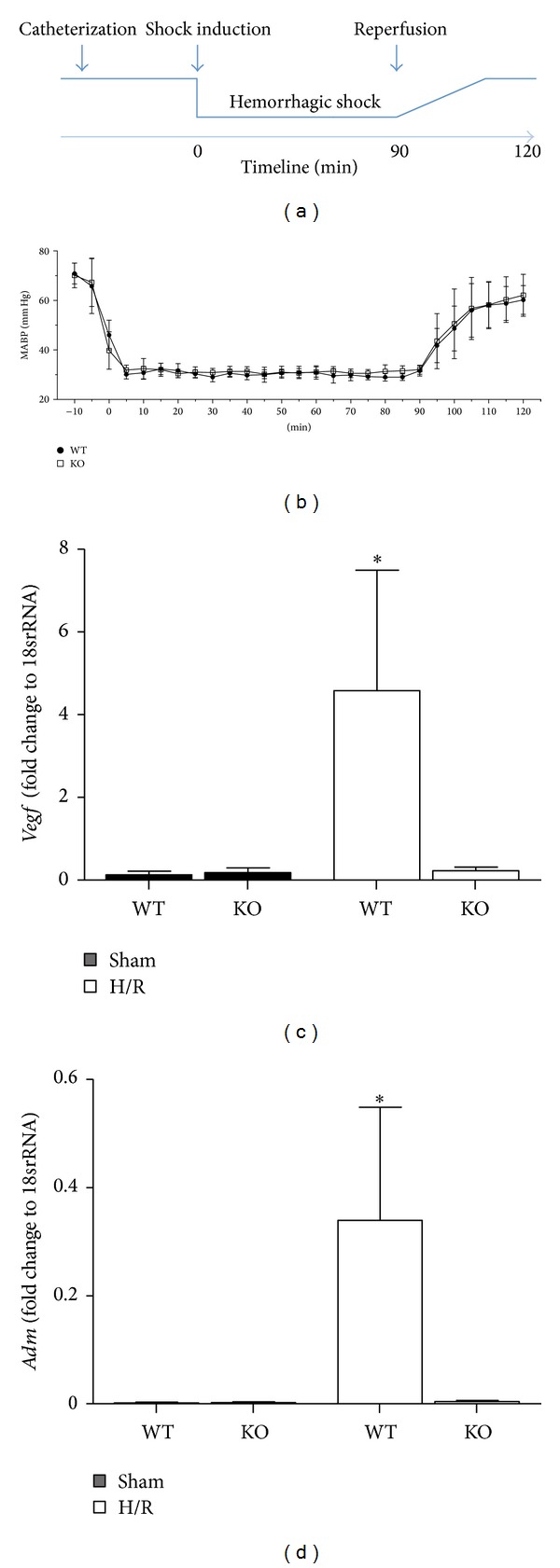
Schematic timeline of the experimental design (a). Blue line denotes the mean arterial blood pressure (MABP) before, during, and after hemorrhage and resuscitation. (b) Mean arterial blood pressure was measured before hemorrhagic shock and at different times during the hemorrhage/resuscitation protocol in wild-type (WT, *n* = 8) and HIF-1*α* knockout mice (KO, *n* = 12). (c)-(d) Hepatic gene expressions of* vegf* (c) and* adm* (d) were analyzed. Gene expression was measured as fold change compared to 18srRNA expression (*P < 0.05 versus all other groups).

To evaluate potential effects of the HIF-1*α* KO on the arterial blood pressure in our model, we measured continuously blood pressure in WT and KO mice before, during, and after H/R. Blood pressure of WT mice was comparable to KO mice during the complete time window used for the measurement (Figure 1(b)). The amount of blood removed to induce and maintain hemorrhagic shock at 30 ± 2 mm Hg was comparable in both groups (0.56 ± 0.05 and 0.58 ± 0.04 mL in WT and KO mice, resp.). These data suggest that the HIF-1*α* KO did not influence the blood pressure either before, during, or after resuscitation.

### 3.2. Analysis of Hepatic Gene Expression of HIF-1*α*-Regulated Genes after Hemorrhage and Resuscitation

The semiquantitative real-time PCR showed an increase in* Vegf* and* Adm* gene expression at 6 h after resuscitation in liver samples obtained from WT mice undergoing H/R as compared to all other groups (*P* < 0.05, Figures 1(c) and 1(d)). This increase was not observed in HIF-1*α* KO mice after H/R (Figures 1(c) and 1(d)).

These results indicate that in the KO mice the HIF-1*α* knockout is of functional significance and verify the model used here.

### 3.3. Cell Damage after Hemorrhage and Resuscitation

**Figure 2:**
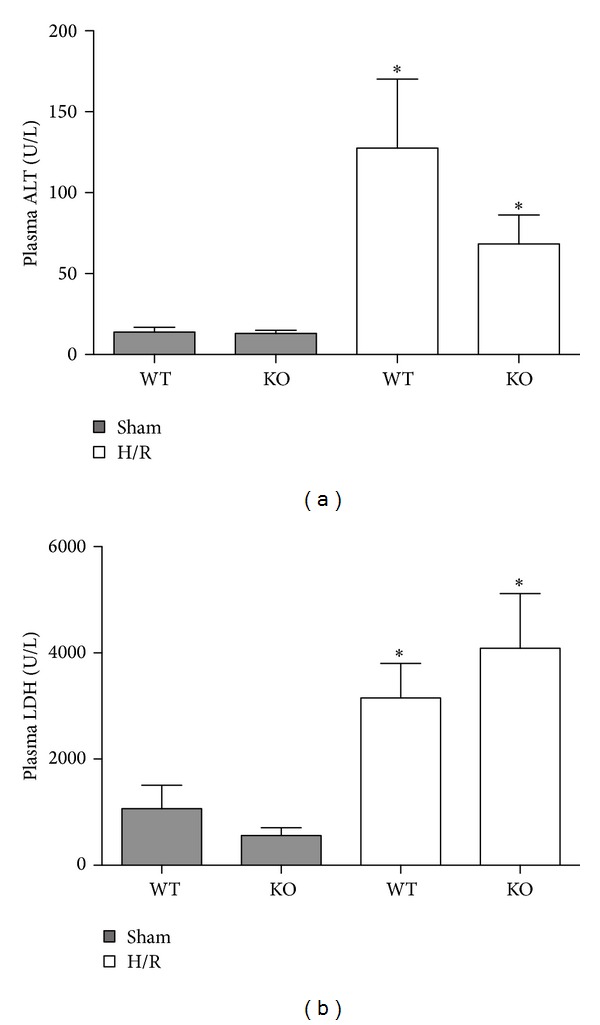
Plasma ALT (a) and LDH (b) levels after hemorrhage and resuscitation in rats. Blood was collected at 6 h after resuscitation for measurement of enzyme levels. WT denotes wild-type mice; KO denotes HIF-1*α* knockout mice prior to sham operation (sham) or hemorrhagic shock with subsequent resuscitation (*P < 0.05 versus both sham groups).

H/R induced a significant increase of plasma ALT, a marker of hepatocellular damage, at 6 h after H/R to 127.6 ± 113.0 IU/L in WT mice and in HIF-1*α* KO mice to 68.0 ± 59.0 IU/L (*P* > 0.05, Figure 2(a)) as compared to sham-operated mice (WT: 13.6 ± 7.5 and KO: 13 ± 5.1 IU/L, resp., Figure 2(a), *P* < 0.05 compared to both H/R groups).

LDH, an indicator of general cell damage, rose up to 3151.0 ± 1730.0 (WT) and 4088.0 ± 3400.0 (KO) IU/L 6 h after resuscitation as compared to sham-operated mice from both groups (WT: 1067.0 ± 1083.0 and KO: 562.0 ± 361.0 IU/L, resp., *P* < 0.05, Figure 2(b)).

HIF-1*α* KO did decrease significantly neither ALT nor LDH values after H/R as compared to WT mice (Figures 2(a) and 2(b)).

### 3.4. Histopathological Changes in Hepatic Tissue after Hemorrhage and Resuscitation

**Figure 3:**
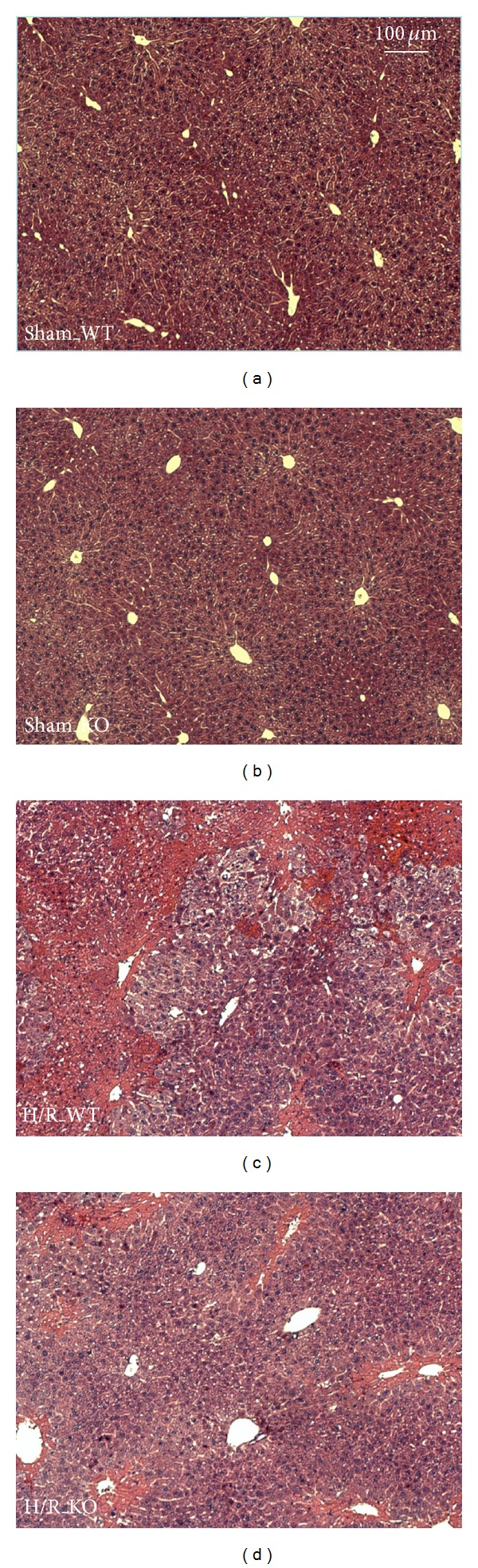
Histological liver injury following hemorrhagic shock and resuscitation (H/R). Sham-operated animals underwent the same surgical procedures but H/R was not carried out. WT denotes wild-type mice; KO denotes HIF-1*α* knockout mice. Representative hematoxylin and eosin stained liver lobes from WT ((a) after sham operation and (c) 6 h after resuscitation, resp.) and KO mice ((b) after sham operation and (d) 6 h after H/R).

Liver sections from WT mice (Figure 3, left row) revealed areas of coagulative necrosis at 6 h after H/R (Figure 3(c)). In HIF-1*α* KO mice (Figure 3, right row), hepatocytes with cellular enlargement and nuclear dissolution were also highly present after H/R (Figure 3(d)). KO mice and WT show comparable amount of cell damage. These changes were not detected after sham operation (Figures 3(a) and 3(b)).

### 3.5. Systemic and Local Proinflammatory Changes after Hemorrhage and Resuscitation: Plasma and Hepatic IL-6 Levels

**Figure 4:**
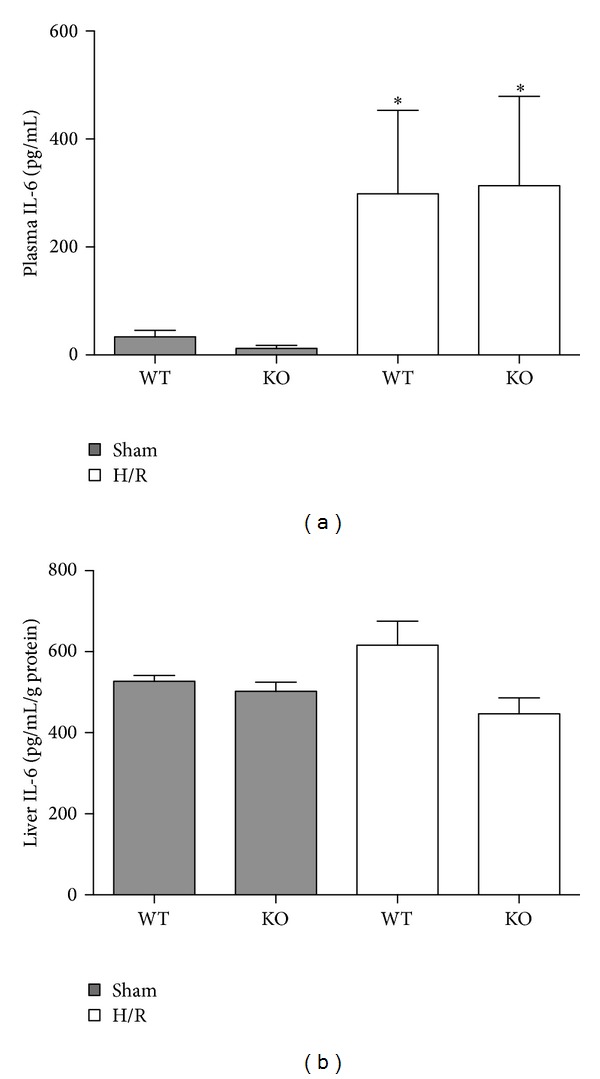
Plasma IL-6 levels (a) and liver IL-6 protein content (b) following hemorrhagic shock and resuscitation (H/R). WT denotes wild-type mice; KO denotes HIF-1*α* knockout mice prior to sham operation (sham) or 6 h after H/R (*P < 0.05 versus both sham groups).

Hemorrhage followed by resuscitation induces a systemic and local immune response, which was determined at 6 h after H/R by plasma and hepatic IL-6 levels. Compared to both sham groups (WT: 33.0 ± 12.0 and KO: 11.0 ± 6.0 pg/mL), plasma IL-6 levels increased significantly in both H/R groups at 6 h after resuscitation (WT: 299.0 ± 154.0 and KO: 313.0 ± 165.0, *P* < 0.05 versus sham group, Figure 4(a)). After H/R hepatic IL-6 levels were slightly but not significantly enhanced in WT compared to KO mice (Figure 4(b)). Nevertheless, the IL-6 levels did not markedly increase compared to sham groups (Figure 4(b)).

These results showed that HIF-1*α* KO cannot prevent systemic proinflammatory changes occurring after H/R in our model.

### 3.6. Hypoxia in Hepatic Tissue after Hemorrhage and Resuscitation

**Figure 5:**
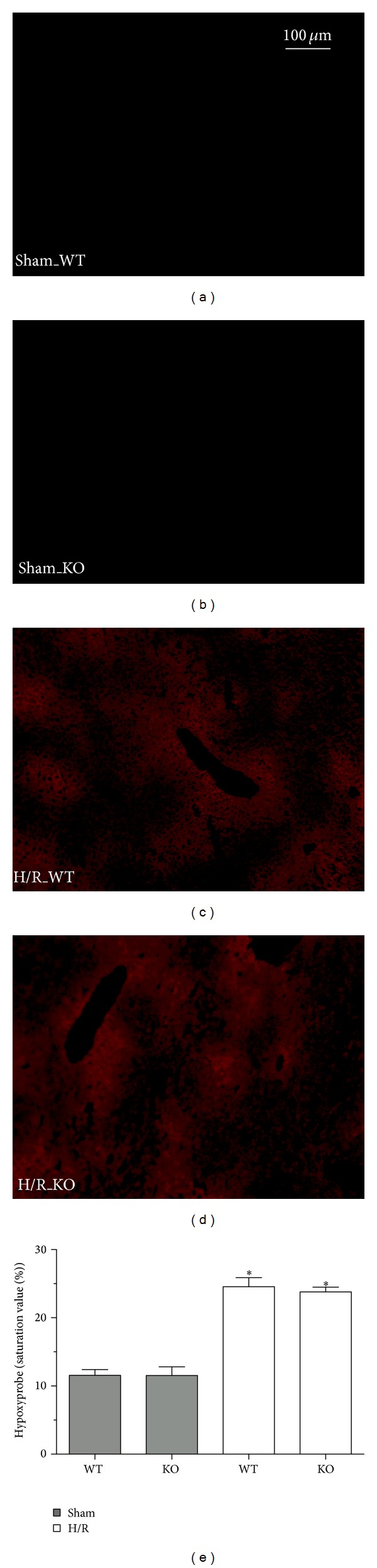
Hepatic hypoxia was assessed by immunohistological staining of Hypoxyprobe-1 as the marker of tissue ischaemia (red (a)–(d)). Sham-operated animals underwent the same surgical procedures but hemorrhagic shock and resuscitation (H/R) was not carried out. Representative liver sections from wild-type (WT) mice ((a) after sham operation and (c) 6 h after resuscitation, resp.) and HIF-1*α* knockout (KO) mice ((b) after sham operation and (d) 6 h after H/R) are shown. Bar is 100 *μ*m. Semiquantitative evaluation of hypoxic tissue revealed the extent of hypoxia (e) (*P < 0.05 versus both sham groups).

Histopathologic evaluation of hypoxic tissue revealed a significant expression of pimonidazole protein (Hypoxyprobe-1) as the marker of tissue ischaemia at 6 h after reperfusion in both WT and HIF-1*α* KO mice as compared to both sham groups (*P* < 0.05, Figure 5). The enhanced pimonidazole protein levels after H/R were comparable between the WT and HIF-1*α* KO group (*P* > 0.05, Figure 5(d)). In sham groups there was no significant expression of pimonidazole protein in the liver tissue (Figure 5).

These results indicate comparable severity of hypoxia in both WT and HIF-1*α* KO mice.

### 3.7. Analysis of the NF-*κ*B Phosphorylation after Hemorrhagic Shock and Resuscitation

**Figure 6:**
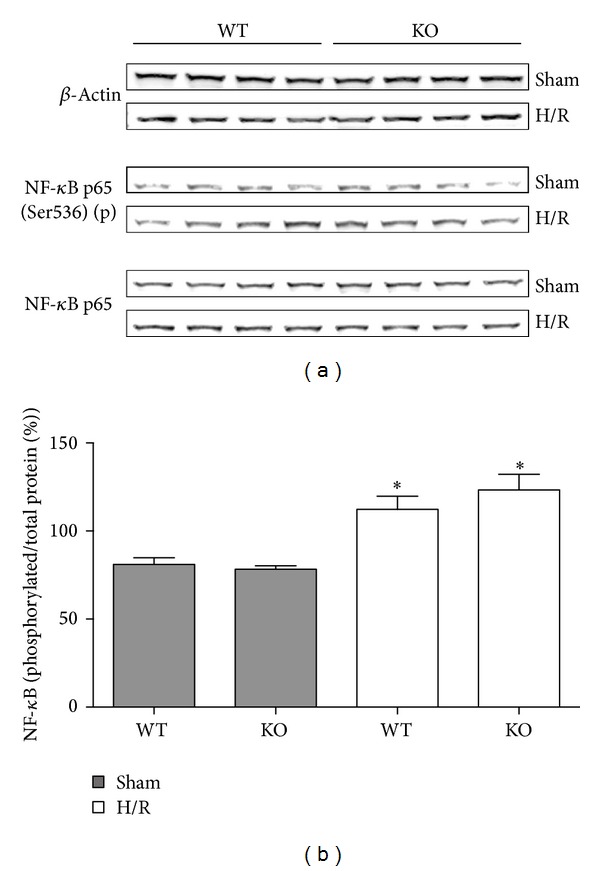
Wild-type (WT) or HIF-1*α* knockout (KO) mice were subjected to hemorrhagic shock and resuscitation (H/R) or sham operation. Six hours after the end of resuscitation, liver tissue was harvested and western blot for the phosphorylated or nonphosphorylated p65 subunit of NF-*κ*B and *β*-actin was performed (a). Lines 1, 3, and 5 were liver protein extracts from mice after sham operation, and lines 2, 4, and 6 from H/R mice (lanes 1–4: WT, lanes 5–8: KO). Representative gel from 3 experiments is shown. In (b), the ratio of phosphorylated p65 subunit of NF-*κ*B and total protein after densitometric measurements and normalization to *β*-actin is represented (*P < 0.05 versus both sham groups).

To analyze the questionable key role of either HIF-1*α* or NF-*κ*B in our model of acute inflammation, detection of phosphorylated and nonphosphorylated NF-*κ*B in WT and HIF-1*α* KO mice was performed by western blot in liver homogenates collected at 6 h after resuscitation (Figure 6). The relative protein levels of NF-*κ*B indicate similar protein levels in both WT and HIF-1*α* KO mice and in sham and H/R mice (Figure 6). The relative protein levels of phosphorylated NF-*κ*B indicate hardly detectable protein levels in sham groups. The relative protein levels of phosphorylated NF-*κ*B were slightly enhanced after H/R in both WT and HIF-1*α* KO mice (Figure 6). The expression levels of phosphorylated NF-*κ*B seem comparable between WT and HIF-1*α* KO mice after H/R. The quantification of NF-*κ*B expression revealed that the increase in NF-*κ*B phosphorylation after H/R compared to sham groups was significant in both WT and KO mice (Figure 6(b)).

These results indicate that H/R-induced NF-kappaB phosphorylation in the liver is not prevented by the HIF-1*α* KO in the myeloid cells of the liver.

## 4. Discussion

In the present study we examined the role of HIF-1*α* in myeloid cells on general liver injury and proinflammatory changes induced by hemorrhage followed by resuscitation and also provide indication for the important role of NF-*κ*B activation in our model. H/R induced hypoxia, hepatic injury, and release of systemic proinflammatory cytokine IL-6 (Figures 2–5). These changes were comparable in both WT and HIF-1*α* KO mice and seem associated with NF-*κ*B phosphorylation, suggesting that the activation of NF-*κ*B or other inflammation modulators is involved in the pathogenesis of liver damage induced by H/R rather than by HIF-1*α* (Figure 6).

HIF-1*α* is a transcription factor that regulates not only numerous genes involved in erythropoiesis, glycolysis, iron metabolism, and cell survival but also the primary cellular response to hypoxia or ischemia [32]. It can be induced by cytokines, bacteria, LPS, and/or hypoxia [33, 34]. Among the various genes induced by HIF-1 is also the induction of NF-*κ*B [35].

NF-*κ*B regulates numerous inflammatory mediators such as IL-6, TNF*α*, and heme oxygenase-1, which are clearly involved in the pathogenesis of liver damage induced by H/R [36, 37]. NF- *κ*B consisting of two subunits, that is, p65 and p50, is bound to its inhibitory protein I*κ*B and thereby maintained in the cytoplasm [38]. Phosphorylation of I*κ*B induces its proteolytic degradation enabling NF-*κ*B to translocate into the nucleus and activate its target genes [38–40]. Various studies have reported NF-*κ*B activation in different tissues, lung, heart, and liver after hemorrhagic shock and subsequent resuscitation [24, 41]. In our previous studies, we found that NF-*κ*B inhibition in the setting of H/R suppresses the proinflammatory response and liver damage [30, 31]. Consistent with these findings, we detect enhanced levels of phosphorylated p65 subunit at six hours after resuscitation in the liver. This increase was present in both WT and HIF-1*α* KO mice indicating an activation of NF-*κ*B that was independent of the HIF-1*α* KO in the myeloid cells at this particular time in our model of H/R. Moreover, consistent with previous findings, we detected enhanced systemic IL-6 levels after H/R [12, 30]. The important role of IL-6 in H/R-induced liver injury was demonstrated in IL-6 KO mice that were protected from hepatic injury [21]. Consistent with previous observations, enhanced systemic IL-6 levels seem to be associated with p65 phosphorylation. Interestingly, HIF-1*α* KO in the myeloid cell-line did not reduce plasma IL-6 levels after H/R in our study. Given the increased* Vegf* and* Adm* gene expressions after H/R in WT mice that were not observed in HIF-1*α* KO mice, these data demonstrate the relevance of the knockout on the transcriptional level and suggest that the early proinflammatory response is rather induced by NF-*κ*B or some other factors than by HIF-1*α* in myeloid cells. However, to address the contribution of the myeloid HIF-1*α* KO in our H/R model regarding VEGF, serum levels of VEGF were determined as well, showing that low VEGF levels are present in the circulation of both WT and KO mice (data not shown). There appeared a trend to an increase of VEGF in serum after H/R in WT and KO mice, demonstrating that the KO in the myeloid cells does not significantly modulate serum values of VEGF after H/R in our model (data not shown). This data demonstrates that the local alterations of* vegf* in the liver are rather due to the HIF-1*α* KO in the myeloid cells. However, the local VEGF levels may not be representative for the systemic changes or changes of VEGF in other tissues. These findings indicate that there are other key factors in the modulation of the inflammation after H/R. Interestingly, a recent study demonstrated that the local cardiomyocyte IL-6 release after trauma hemorrhage was HIF-1*α* mediated [42]. Here, the relevance of the HIF-1*α* KO in the myeloid cell-line for the local hepatic inflammation was not evident due to only slightly increased levels of IL-6 in the WT group after H/R (Figure 4(b)). Previously, we demonstrated enhanced local hepatic IL-6 in the model of H/R in rats [30]. In principle, investigating a single fixed time for sacrifice is not reliable enough to exclude the potential role of HIF-1*α* in H/R. However, the observed effects on systemic IL-6 increase and NF-*κ*B phosphorylation associated with hepatic damage in both WT and HIF-1*α* KO mice after H/R indicate NF-*κ*B significance in this model but reveal as well the need for further studies to uncover important key players and mechanisms within this setting. Recent studies provide evidence of hypoxic HIF-independent inflammation and NF-*κ*B coactivation [43–45]. Other reports indicate a close interference between NF-*κ*B and HIF and demonstrate their crosstalk as responsible element for basal HIF-1*α* gene expression [27, 28]. On the other hand, HIF overexpression interferes with NF-*κ*B signaling by increasing NF-*κ*B activity and gene expression [29]. Although HIF-1*α* was not present in the myeloid cell-line in our KO mice, p65 subunit of NF-*κ*B was phosphorylated after H/R. These data indicate that either HIF-1*α*-independent NF-*κ*B activation or the p65 subunit phosphorylation has been shown in hepatocytes and not in myeloid cells. Whether the HIF-1*α* KO influenced the NF-*κ*B activation in myeloid cells has not been answered yet.

In conclusion, NF-*κ*B plays an important role in the pathogenesis of hepatic injury after H/R by upregulation of systemic IL-6. HIF-1*α* knockout in myeloid cell-line could not prevent the systemic IL-6 release and liver injury or the phosphorylation of p65 after H/R. Hence, our results suggest that there are other essential factors involved in the pathogenesis of H/R-induced liver injury. Furthermore, the data indicate that NF-*κ*B presumably in the liver rather than HIF-1*α* in myeloid cells may act with causative character for hepatic injury after H/R.
